# Ontogenetic Variation in the Mineral, Phytochemical and Yield Attributes of Brassicaceous Microgreens

**DOI:** 10.3390/foods10051032

**Published:** 2021-05-10

**Authors:** Marios C. Kyriacou, Christophe El-Nakhel, Antonio Pannico, Giulia Graziani, Armando Zarrelli, Georgios A. Soteriou, Angelos Kyratzis, Chrystalla Antoniou, Fabiana Pizzolongo, Raffaele Romano, Alberto Ritieni, Stefania De Pascale, Youssef Rouphael

**Affiliations:** 1 Department of Vegetable Crops, Agricultural Research Institute, 1516 Nicosia, Cyprus; m.kyriacou@ari.gov.cy (M.C.K.); soteriou@ari.gov.cy (G.A.S.); A.Kyratzis@ari.gov.cy (A.K.); chrystalla.antoniou@ari.gov.cy (C.A.); 2 Department of Agricultural Sciences, University of Naples Federico II, 80055 Portici, Italy; christophe.elnakhel@unina.it (C.E.-N.); antonio.pannico@unina.it (A.P.); fabiana.pizzolongo@unina.it (F.P.); raffaele.romano@unina.it (R.R.); depascal@unina.it (S.D.P.); 3 Department of Pharmacy, University of Naples Federico II, 80131 Naples, Italy; giulia.graziani@unina.it (G.G.); alberto.ritieni@unina.it (A.R.); 4 Department of Chemical Sciences, University of Naples Federico II, 80126 Naples, Italy; zarrelli@unina.it

**Keywords:** antioxidants, bioactive value, carotenoids, harvest maturity, isothiocyanates, ontogeny, polyphenols, volatile organic compounds

## Abstract

Microgreens constitute novel gastronomic ingredients that combine visual, kinesthetic and bioactive qualities. The definition of the optimal developmental stage for harvesting microgreens remains fluid. Their superior phytochemical content against mature leaves underpins the current hypothesis of significant changes in compositional profile during the brief interval of ontogeny from the appearance of the first (S1) to the second true leaf (S2). Microgreens of four brassicaceous genotypes (Komatsuna, Mibuna, Mizuna and Pak Choi) grown under controlled conditions and harvested at S1 and S2 were appraised for fresh and dry yield traits. They were further analyzed for macro- and micromineral content using inductively coupled plasma optical emission spectrometry (ICP-OES), carotenoid content using high-performance liquid chromatography with a diode-array detector (HPLC-DAD), volatile organic compounds using solid-phase microextraction followed by gas chromatography-mass spectrometry (SPME-GC/MS), anthocyanins and polyphenols using liquid chromatography-high resolution-tandem mass spectrometry (LC-MS/MS) with Orbitrap technology and for chlorophyll and ascorbate concentrations, well as antioxidant capacity by spectrophotometry. Analysis of compositional profiles revealed genotype as the principal source of variation for all constituents. The response of mineral and phytochemical composition and of antioxidant capacity to the growth stage was limited and largely genotype-dependent. It is, therefore, questionable whether delaying harvest from S1 to S2 would significantly improve the bioactive value of microgreens while the cost-benefit analysis for this decision must be genotype-specific. Finally, the lower-yielding genotypes (Mizuna and Pak Choi) registered higher relative increase in fresh yield between S1 and S2, compared to the faster-growing and higher-yielding genotypes. Although the optimal harvest stage for specific genotypes must be determined considering the increase in yield against reduction in crop turnover, harvesting at S2 seems advisable for the lower-yielding genotypes.

## 1. Introduction

Microgreens have gained a position over the past two decades initially in the upscale gastronomy market and subsequently in the mainstream horticultural supply chain as ingredients of outstanding gastronomic and nutritive value. They combine visual, kinesthetic and bioactive qualities derived from their rich mineral and phytochemical content. The range of genotypes (species, cultivars, landraces) employed for growing microgreens commercially has been rapidly expanding along with the industry, yet the majority of genotypes come from the families *Brassicaceae*, *Asteraceae*, *Chenopodiaceae*, *Lamiaceae, Apiaceae*, *Amarillydaceae, Amaranthaceae* and *Cucurbitaceae* [1]. 

Microgreens have been shown to be good sources of minerals that constitute necessary dietary intake requirements, particularly of the macroelements (K, Ca and Mg) and the microelements (Fe and Zn; [2,3]). The phytochemical content of microgreens attributed bioactive value comprises phenolic compounds, encountered as flavonoids (including anthocyanidins) and nonflavonoids (mostly phenolic and hydroxycinnamic acids), carotenoids (β-carotene, lutein and violaxanthin), ascorbic acid, phylloquinone and tocopherols [4,5,6,7]. Microgreens of the *Brassicaceae*, which comprise the most extensive repository of genetic resources for commercial microgreens production [1,8], are also significant sources of glucosinolates [9,10], encountered mostly in the form of isothiocyanates [11], which indicates an active glucosinolate metabolism in the early post-germination stages of development. 

The mineral and phytochemical composition of microgreens and their *in vitro* antioxidant capacity has been found responsive to several preharvest factors, among which the most influential seems to be the choice of genotype [1,4]. Growth conditions nonetheless also demonstrate the capacity to modulate the compositional profiles of microgreens significantly. These include substrate material, which may range considerably in physicochemical parameters between natural fiber and synthetic substrates [3,12], and nutrient supplementation strategies [13,14,15,16,17]. Moreover, pre-harvest light conditions (quality, intensity and photoperiod) have been demonstrated effective tools in modulating growth and compositional attributes of microgreens, particularly secondary metabolites, rendering Light Emitting Diode (LED) light modules instrumental for the operation of microgreen plant factories [18,19,20].

The developmental stages of the early post-germination plant ontogeny may dictate significant changes in plant physicochemical constitution and phytochemical content [6,21,22]. The optimal stage for harvesting microgreens should combine prime yield attributes and high crop turnover with prime sensory and functional quality. Previous research on microgreens differentiates clearly sprouts from microgreens, based on light requirements during growth and the harvesting of microgreens without roots at the substrate-hypocotyl interface. Yet the definition of microgreens with respect to the exact developmental stage at harvest remains relatively fluid. Kyriacou et al. [1] defined microgreens as greens harvested upon the appearance of the first pair of true leaves, when cotyledons are fully expanded and still turgid. However, the developmental stage at harvest reported in different works varies, with some applying harvest at the cotyledonary stage [23,24], others at the appearance of the first true leaf [6,8,25] and yet others at the second true leaf [3,7,24,26]. Other than the obvious change in fresh biomass between successive stages of ontogeny, previous work has also demonstrated significant changes in secondary plant metabolites and mineral content that constitute integral parts of the vegetables’ functional qualities [27].

The superior phytochemical content of microgreens against mature leaves postulated in previous works [4,21,24], has underpinned the current hypothesis that significant changes in compositional profile may take place during the brief interval of microgreens’ ontogeny from the appearance of the first (S1) to the second true leaf (S2). Accordingly, microgreens of four brassicaceous genotypes (Komatsuna, Mibuna, Mizuna and Pak Choi) grown under controlled conditions and harvested at S1 and S2 were appraised for fresh and dry yield traits. They were further analyzed for macro- and micromineral content by inductively coupled plasma optical emission spectrometry (ICP-OES), carotenoid content through HPLC-DAD, volatile organic compounds using SPME-GC/MS, anthocyanins and polyphenols using Orbitrap LC-MS/MS and chlorophyll and ascorbate concentrations as well as in vitro antioxidant capacity by spectrophotometry. The results of the current study will enhance our understanding of potential ontogenetic variation in the mineral, phytochemical and yield attributes of brassicaceous microgreens and therefore, in the elucidation of microgreens definition with respect to harvest maturity.

## 2. Materials and Methods

### 2.1. Microgreen Genotypes and Growth Chamber Conditions

Two green-pigmented genotypes [(mibuna (*Brassica rapa* L. subsp. *nipposinica*) and mizuna (*Brassica rapa* var. *japonica* cv. Greens)] and two red-pigmented genotypes [komatsuna (*Brassica rapa* L. var. *perviridis*) and Pak Choi (*Brassica rapa* L. subsp. *chinensis*)] were used. Mizuna seeds were provided by Condor Seed Production (Yuma, AZ, USA), while the rest of the seeds were bought from CN Seeds Ltd (Pymoor, Ely, Cambrigeshire, UK). The experiment was carried out at the Agricultural Research Institute (Department of Vegetable Crops), Nicosia, Cyprus, in a controlled growth chamber (MIR-554 growth chamber, Panasonic, Gunma, Japan). Seeds were sown at a density of 70,000 seeds m^−2^ and germinated in darkness at 24 °C and 100% relative humidity (RH), by applying irrigation with osmotic water until emergence. Upon emergence, the growth chamber was set at 24/18 ± 2 °C and RH at 65/75% according to a 12 h photoperiod delivered by Light Emitting Diode (LED) panels (K5 Series XL750, Kind LED, Santa Rosa, CA, USA). Photosynthetic photon flux density at the canopy level was maintained at 300 ± 10 μmol m^−2^ s^−1^. Growth was facilitated in plastic trays (W × L × D: 14 × 19 × 6 cm) filled with a peat-based substrate, as described in detail by Pannico et al. [28]. Daily fertigation with a quarter-strength modified Hoagland formulation (electrical conductivity 400 ± 50 mS cm^−1^ and pH 6 ± 0.2) was applied using a laboratory beaker. The composition and concentration of the constituent macro- and microelements is detailed in Pannico et al. [28]. All treatments were replicated three times while daily rotation of tray positions maintained an equal distribution of light and humidity over the chamber growth surface.

### 2.2. Canopy Colorimetric Measurements, Phenological Harvest Stage and Sampling

**Figure 1:**
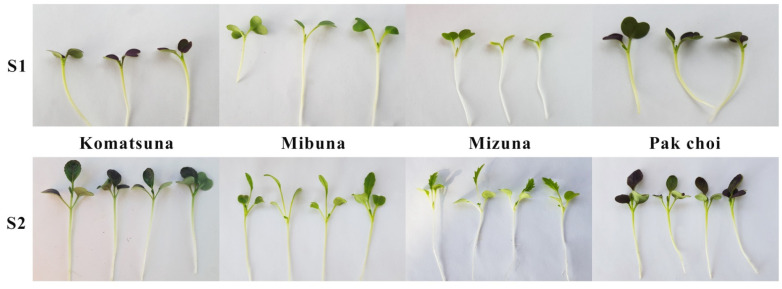
Phenological stages for harvesting four *Brassica rapa* L. genotypes (Komatsuna, Mibuna, Mizuna, Pak Choi grown under controlled conditions: S1-appearance of the first true leaf; S2-appearance of the second true leaf).

Prior to microgreen harvesting, CIELAB color space chroma of the microgreens canopy was measured by means of a portable chroma meter (CR-400, Minolta, Osaka, Japan). Eight measurements per tray/replicate were obtained. Microgreens of the four cultivated Brassica genotypes were harvested at two different growth stages (Figure 1): S1 (appearance of the first true leaf) and S2 (appearance of the second true leaf). S1 and S2 in all genotypes were attained 7 and 12 days after sowing, respectively. At each stage, microgreens were harvested just above substrate level and immediately assessed for fresh weight. Afterwards, a random part of the sample was used fresh for the analysis of volatile organic compounds by SPME-GC/MS, another part was conserved at −80 °C for phytochemical analyses (either fresh-frozen or lyophilized), and a part was placed in a forced-air oven at 65 °C until reaching constant dry weight, as determined on an analytical balance (XT120A, Precisa Gravimetrics, Dietikon, Switzerland). Dry matter content was calculated and expressed as percentage of fresh weight. Dried microgreens leaves and stems were ground in a Wiley Mill (841-microns screen) for mineral analysis. 

### 2.3. Chlorophyll, Total Ascorbic Acid and Hydrophilic Antioxidant Capacity

The Lichtenhaler and Wellburn [29] protocol was implemented for determining the chlorophyll content of microgreens. Spectrophotometric determination of chlorophylls *a* and *b* extracted in 90% acetone was performed at 662 and 645 nm on a Hach DR 4000 spectrophotometer (Hach Co., Loveland, CO, USA). Total chlorophyll content was calculated as the sum of chlorophylls *a* and *b* and expressed as μg kg^−1^ fw. 

Analysis of total ascorbic acid (TAA) was performed according to the method of Kampfenkel et al. [30] and the results were expressed in mg kg^−1^ fw. The hydrophilic antioxidant capacity (HAC) of lyophilized microgreens was assessed through UV-Vis spectrophotometry based on the *N*,*N*-dimethyl-p-phenylenediamine (DMPD) method of Fogliano et al. [31] and expressed in mmol ascorbate equivalents kg^−1^ dw.

### 2.4. Analysis of Macro- and Micro-Minerals by ICP-OES

Macro (P, K, Ca, Mg, and Na) and microminerals (Fe, Mn, Mo, Se and Zn) were determined using inductively coupled plasma optical emission spectrometry (ICP-OES Spectroblue, Spectro Ametek, Berwyn, PA, USA), as detailed in Volpe et al. [32]. Microwave-assisted digestion (MLS-1200 Microwave Laboratory Systems, Milestone, Shelton, CT, USA) of about 1 g of desiccated microgreens in 10 mL of 65% HNO_3_ and 37% HCl (3:1, *v*/*v*) was performed and the digested solutions were then brought to 50 mL with ultrapure water (Milli-Q, Merck Millipore, Darmstadt, Germany). Calibration curves for nonalkaline elements (Fe, Mn, Mo, Se and Zn) were established in the range of standard concentrations from 1.0 to 100 μg L^−1^ and expressed in μg g^−1^ dw. Calibration curves for alkaline elements (P, K, Ca, Mg, and Na) were established in the range of standard concentrations from 100 μg L^−1^ to 10 mg L^−1^ and expressed in mg g^−1^ dw. Accuracy was checked by concurrent analysis of standard reference material (BCR CRM 142R-Commission of the European Communities, 1994) and recoveries ranged from 86 to 98%.

### 2.5. Analysis of Carotenoids by HPLC-DAD

Carotenoids from lyophilized microgreens samples were extracted, separated and quantified as previously described by Kyriacou et al. [7]. An Agilent HPLC system (Agilent Technologies, Santa Clara, CA, USA) furnished with a 1200 Series quaternary pump and a 1260 Diode Array Detector Separation was employed. Separation was implemented on a Gemini C18 (Phenomenex, Torrance, CA, USA) reverse phase column (250 mm × 4.6 mm, 5 μm). Quantification was based on external standard calibration curves established using β-carotene and lutein commercial standards at six concentrations in the range 5 to 100 μg mL^−1^. Results were expressed in mg k^−1^ dw. A representative HPLC-Diode array chromatogram of carotenoids extracted from Komatsuna microgreens monitored at 450 nm is reported in Figure S1.

### 2.6. Analysis of Anthocyanins and Polyphenols by UHPLC-Q-Orbitrap HRMS

Anthocyanins and polyphenols were extracted from lyophilized microgreens and analyzed by the method detailed in Kyriacou et al. [7] and El-Nakhel et al. [33]. Analysis was facilitated on a UHPLC system (Dionex UltiMate 3000, Thermo Fisher Scientific, MA, USA) coupled to a Q-Exactive Orbitrap mass spectrometer (UHPLC, Thermo Fischer Scientific, MA, USA). Chromatographic separation of anthocyanins and polyphenols was performed using a Luna Omega PS 1.6 µm (50 mm × 2.1 mm, Phenomenex) column. Compound identification and quantification was facilitated using a Q-Exactive Orbitrap mass spectrometer (UHPLC, Thermo Fischer Scientific, Waltham, MA, USA) operated in fast negative/positive ion switching mode (Thermo Scientific, Bremen, Germany). Two scan events (Full scan MS and All ion fragmentation, AIF) were set for all compounds of interest. Analysis and processing of data was performed using software Xcalibur v.3.0.63 (Xcalibur, Thermo Fisher Scientific, Waltham, MA, USA). 

The concentrations of all anthocyanin and polyphenol compounds were expressed in µg g^−1^ dw. Representative UHPLC-HRMS chromatograms of anthocyanin and polyphenol compounds extracted from Komatsuna microgreens are reported in Figures S2 and S3. The individual phenolic compounds were identified and quantified by comparison with available standards. In particular, anthocyanins were quantified using an external calibration curve built with cyanidin-glucoside and their concentration was expressed as cyanidin-glucoside equivalents. As regards polyphenolic compounds the external calibration curves of the following standards have been used: rutin for all quercetin derivatives, kaempferol-3-*O*-glucoside for all kaempferol derivatives, caffeic acid for caffeic acid and its derivatives, ferulic acid for ferulic acid and its derivatives, apigenin-7-*O*-rutinoside for apigenin-7-*O*-rutinoside and its derivative, naringin for naringin and finally luteolin-3-*O*-rutinoside reference standard for luteolin-3-*O*-rutinoside. The robustness of the analytical method was assessed using the corresponding standards. As some standards were not available for identification MS/MS experiments had to be used. Linearity, the limit of detection (LOD), limit of quantification (LOQ), precision and recovery for the eight authentic standards were reported in Supplementary Material (Table S1).

### 2.7. Analysis of Volatile Organic Compounds by SPME-GC/MS

Volatile organic compounds (VOCs) were extracted by Solid-Phase Micro-Extraction (SPME) and analyzed by Gas Chromatography coupled to Mass Spectrometry (GC/MS), following the methodology outlined by Huang et al. [34] and Klimankova et al. [35], with some modifications. One g of fresh microgreens was positioned in a 20 mL headspace glass vial with a screw-top PTFE septum (Supelco^®^, Bellefonte, PA, USA). Migration of the VOCs to headspace was induced by 10 min stirring at 40 °C with an ARE^®^ magnetic stirrer (Velp^®^ Scientifica, Usmate Velate, Monza Brianza, Italy). Adsorption of VOCs was implemented by introducing a 50/30-μm-thick, one-cm-long divinylbenzene/carboxane/polydimethylsiloxane fiber into the vial (Supelco^®^, Bellefonte, PA, USA) for 20 min at 40 °C. The fiber was subsequently injected for desorption into a split-splitless injector at 230 °C for 10 min of an Agilent 6890N GC coupled to a 5973N MS detector (Agilent, Santa Clara, CA, USA). Following a splitless injection of the samples, separation was facilitated in a capillary column 30-m-long and 0.250-mm-thick, coated with a 0.25-μm film of 5% phenyl/95% dimethylpolysiloxane (Supelco^®^, Bellefonte, PA, USA). Oven temperature was kept at 50 °C for 2 min, then ramped from 50 to 150 °C at 10 °C min^−1^ and from 150 to 280 °C at 15 °C min^−1^. Helium was used as the carrier gas at 1 mL min^−1^. Injection source and ion source temperatures were 250 and 230 °C, respectively, and the MS was operated at 70 eV. Headspace VOCs were analyzed by comparing mass spectra and retention times with the Atomic Spectra Database version 1.6 of National Institute of Standards and Technology (NIST) libraries. Samples were analyzed in triplicates and the results were expressed in percentage (%). Representative chromatograms of VOCs extracted by SPME and analyzed by GC/MS, from Komatsuna and Pack Choi microgreens are reported in Figure S4.

### 2.8. Statistical Analysis

Data were subjected to two-way analysis of variance (ANOVA) using JMP statistical package (SAS Institute, Inc., Cary, NC, USA). Genotype means and genotype × growth stage means were compared and separated according to the Tukey–Kramer honestly significant difference (HSD) test. Growth stage means were compared according to the Student’s *t*-test, provided that the main effect of the growth stage was significant according to the ANOVA.

## 3. Results

### 3.1. Yield, Dry Matter and Colorimetric Components

The highest fresh yield was obtained from Mibuna and Komatsuna microgreens and the lowest from Mizuna (Table 1). A significant increase in fresh yield was observed between growth stages in all genotypes. However, the relative increase in fresh yield between S1 and S2 differed markedly between genotypes, as indicated by significant Μ × S interaction. Intriguingly, the largest increase was noted in Mizuna (80.1%) and Pak Choi (33.3%), which were the genotypes having lower mean yield than the Mibuna and Komatsuna microgreens. Similar effects were observed for dry weight with significantly higher dry weight obtained for Mibuna and Komatsuna than Pak Choi and Mizuna, with the latter incurring however the largest increase (119%) between growth stages. Pak Choi and Mizuna also attained lower dry matter content than Mibuna and Komatsuna. Dry matter increased from S1 to S2 by a mean increment of 12.2% in all genotypes except Mibuna, which did not incur significant change in dry matter content. Chroma was most intense in the green-colored Mibuna and Mizuna compared to the darker-colored Komatsuna and Pak Choi. Chroma increased between S1 and S2 in all genotypes but higher relative increase was observed in the purple-colored than the green-colored genotypes.

### 3.2. Mineral Composition

All eleven macro- and microminerals examined differed significantly between genotypes (Table 2). Pak Choi and Komatsuna were the microgreen genotypes with the highest content in the macrominerals P and K. Pak Choi was also the genotype richest in the macromineral Mg and the microminerals Fe and Zn. Mibuna was the richest genotype in Ca and in the microminerals Mn and Se, whereas Mizuna was the richest in Na and Mo. 

Phosphorous, Na and Mo concentrations did not differ between growth stages in any of the genotypes examined. Potassium, Ca, Mg, Fe, Se and Zn concentrations exhibited a variable response to the growth stage as indicated by significant M × S interaction. Potassium concentration did not differ between stages in Mibuna, Mizuna and Pak Choi, whereas in Komatsuna concentration was lower at S2 compared to S1. Calcium concentration was overall higher at S2 than S1. However, in Mizuna and Komatsuna, this difference was nonsignificant. Magnesium concentration was unchanged between growth stages in Komatsuna, Mizuna and Pak Choi but it increased significantly at S2 in Mibuna. Iron concentration exhibited the most varied response across genotypes with higher concentration observed in Komatsuna and Pak Choi at S1, lower concentration at S1 in Mibuna and no change in Mizuna. Selenium concentration showed no response to the growth stage in all genotypes but Pak Choi demonstrated higher concentration at S1. Finally, Zn concentration increased at S2 in Mizuna but remained unaltered in the rest three genotypes (Table 2). 

In summary, major changes between growth stages were observed for macrominerals K, Ca and Mg and microminerals Fe and Mn. Decrease from S1 to S2 was the prevalent change for the concentrations of K and Fe as opposed to an increase for the concentrations of Ca, Mg, and Mn. Except for Ca, however, these responses were not uniform across genotypes. On the other hand, the concentrations of P, Na, Mo and to lesser degree those of Se and Zn were nonresponsive to the growth stage. 

### 3.3. Antioxidant Capacity, Ascorbic Acid, Chlorophyll, Lutein and β-Carotene

The hydrophilic antioxidant capacity (HAC) of the aqueous extract differed significantly between the four microgreen genotypes studied (Table 3). The HAC was highest in Pak Choi and Komatsuna and lowest in Mizuna. The HAC did not change between growth stages in Mibuna and Pak Choi but was found higher at S1 in Komatsuna and Mizuna. Ascorbic acid concentration also differed significantly between genotypes, being highest in Mizuna and lowest in Pak Choi. Ascorbic acid concentration did not change between growth stages in Mibuna, increased from S1 to S2 in Komatsuna and Mizuna, whereas it decreased from S1 to S2 in Pak Choi. Total chlorophyll content was highest in Komatsuna and Pak Choi, which were additionally the two genotypes of microgreens that demonstrated no change in chlorophyll content between growth stages (Table 3). On the contrary, Mibuna and Mizuna were the two genotypes of microgreens that incurred significant decrease in chlorophyll content from S1 to S2. In all four genotypes, the principal carotenoid concentration was that of β-carotene over lutein, with the former being about two-fold higher than the latter across genotypes (Table 3). Lutein was highest in Mibuna and lowest in Mizuna. Komatsuna, Mizuna and Pak Choi showed no response to the growth stage for lutein concentration whereas Mibuna incurred increased concentrations at S2. Finally, β-carotene was highest in Mibuna and lowest in Mizuna, thus corresponding to the inter-specific differences also observed for lutein. However, β-carotene concentration remained stable between growth stages in all four genotypes of microgreens (Table 3).

### 3.4. Volatile Aromatic Compounds

A total of 18 volatile aromatic compounds (VOCs) were identified in the four microgreen genotypes at the first and second true leaf stages, six of which accounted for more than 90% of the total abundance (total peak area) and each demonstrated a relative abundance above 1% (Table 4). The most abundant VOCs in all four genotypes were the isothiocyanate compounds 3-butenyl isothiocyanate, allyl isothiocyanate and phenethyl isothiocyanate, which accounted for >85% of the VOCs relative abundance. These were succeeded in relative abundance by the aldehydes trans-2-hexenal and nonanal, and the cyclic monoterpene limonene. The relative abundance of all eight major VOCs presented was significantly affected by genotype but presented a variable response to the growth stage, as indicated by significant M × S interaction. 

The highest relative abundance in 3-butenyl isothiocyanate was observed in Mibuna and the lowest in Mizuna; moreover, it was found in higher relative abundance in stage S1 microgreens of all genotypes, except from Mizuna, which demonstrated no change in response to the growth stage (Table 4). A reverse motif was observed for allyl isothiocyanate that was most abundant in Mizuna and least in Mibuna. As opposed to the trend observed for 3-butenyl isothiocyanate, the relative abundance of allyl isothiocyanate increased at S2 in Komatsuna and marginally in Pak Choi but remained nonresponsive to the growth stage in Mibuna and Mizuna microgreens. Pak Choi and Mizuna microgreens had the highest relative abundance of phenethyl isothiocyanate and Mibuna the lowest. In Mizuna and Pak Choi microgreens, the presence of phenethyl isothiocyanate decreased marginally at S2 whereas in Komatsuna it increased and in Mibuna change was nonsignificant (Table 4). Limonene was found in highest relative abundance in Komatsuna and in lowest in Mizuna microgreens. In Mibuna and Pak Choi it increased at S2, whereas Komatsuna and Mizuna demonstrated no change in response to the growth stage. Nonanal was highest in Pak Choi and increased significantly at S2 in Mibuna and Pak Choi whereas in Komatsuna and Mizuna the increase was nonsignificant. The relative abundance of nonanal with response to the growth stage did not change significantly in Komatsuna and Mizuna whereas it increased at S2 in Mibuna and Pak Choi microgreens. 

Finally, trans-2-hexenal was found in highest relative abundance in Pak Choi and in lowest in Mibuna and Mizuna; while it increased at S2 in Pak Choi and Komatsuna, changes the in Mibuna and Mizuna microgreens were nonsignificant (Table 4). Overall, variation in the relative abundance of the major VOCs identified was higher between genotypes than growth stages, with response to the growth stage also varying between genotypes. The most common response to the growth stage was either increase at S2 or no change. It is worth noting however that the most prevalent volatile aromatic component in the microgreens profile (3-butenyl isothiocyanate) had higher relative abundance at S1 than S2, whereas the reverse trend was dominant for the rest of the identified VOCs.

### 3.5. Anthocyanins

Variation in the total concentration of anthocyanins present in the four microgreens genotypes was wide and corresponded distinctly to their visual pigmentation (Table 5). In this respect, total anthocyanins were more concentrated in the purple-leaf genotypes of Pak Choi and Komatsuna and significantly less concentrated in the green-leaf genotypes of Mibuna and Mizuna. The most abundant anthocyanin component in all genotypes was cyanidin-3-(feruloyl)(sinapoyl)dihexoside-5-hexoside, which largely defined genotype behavior in response to the growth stage. The effect of the growth stage on all anthocyanin components examined was nonsignificant (Table 5). However, interspecific differences were observed in response to the growth stage, as highlighted by significant interaction. In the green-leaf genotypes of Mibuna and Mizuna differences between S1 and S2 were nonsignificant for all anthocyanin components, as well as total anthocyanins. For the purple-leaf genotypes of Komatsuna and Pak Choi opposite responses to the growth stage were observed. In Komatsuna, cyanidin-3-(feruloyl)(sinapoyl)dihexoside-5-hexoside, cyanidin-3-(p-coumaroyl)(sinapoyl)dihexoside-5-hexoside as well as total anthocyanins increased at S2 and only cyanidin-3-(caffeoyl)(p-coumaroyl)dihexoside-5-hexoside decreased. In Pak Choi by contrast, cyanidin-3-(feruloyl)(sinapoyl)dihexoside-5-hexoside and total anthocyanins were higher at S1 than S2, while the reverse trend was observed for cyanidin-3-(caffeoyl)(p-coumaroyl)dihexoside-5-hexoside and cyanidin-3-(p-coumaroyl)(sinapoyl)dihexoside-5-hexoside (Table 5). In summary, anthocyanin concentration reflected closely the visual pigmentation of the microgreen species and no response to the growth stage was seen in green-leaf microgreens. Whereas in the case of purple-leaf genotypes response to growth space was genotype-specific with respect to individual anthocyanin components and total anthocyanin content.

### 3.6. Polyphenols

Twenty polyphenols were identified and quantified by UHPLC-Q-Orbitrap HRMS in the four microgreen genotypes at S1 and S2 (Table 6). Genotype effect on all polyphenols and total phenolic content was significant. Growth stage effect was significant most of the phenolic components quantified and the total phenolic content of microgreens with a prevalent trend for increase at S2. However, variation in phenolic content was primarily defined by genotype. Interspecific variation in total phenolic content was wide (630–1933 μg g^−1^ dw), with the highest content observed in Mizuna and the lowest in Komatsuna microgreens (Table 6). Despite differences in total phenolic content however, the four microgreen genotypes presented relatively similar compositional profiles of polyphenols. As a fraction of the total phenolic content across genotypes, flavonol glycosides represented 71.2–82.3%, hydroxycinnamic acids and their derivatives 16.4–26.1% and flavones 1.3–2.7%.

In all genotypes, the most abundant phenolic compounds in declining order were: kaempferol-3-*O*-(synapoyl)-sophoroside-7-*O*-hexoside, quercetin-3-*O*-glucuronide, feruloyl glycoside and caffeic acid hexoside (Table 6). The total phenolic content of microgreens and the phenolic components responsive to the growth stage were higher at S2 than S1. For instance, ferulic acid and feruloyl glycoside concentrations increased in all genotypes from S1 to S2. Therefore, a trend for increasing concentration with transition from S1 to S2 was discerned for most phenolic constituents and the total phenolic content (Table 6). 

## 4. Discussion

### 4.1. Yield Characteristics

The optimal harvest stage for microgreens should combine high yield and high crop turnover while ensuring prime sensory and functional quality. Most available research on microgreens makes a clear differentiation with sprouts based on the harvesting of microgreens without roots at the substrate-hypocotyl interface. However, the exact definition of microgreens with respect to developmental stage at the time of harvest remains relatively vague. In their review on microgreens, Kyriacou et al. [1] defined microgreens as greens harvested upon the appearance of the first pair of true leaves, when cotyledons are fully expanded and still turgid. But the developmental stage at harvest actually varies between different works, with certain researchers harvesting at the cotyledonary stage [23,24], others at the appearance of the first true leaf [6,8,25] and yet others at the second true leaf [3,7,24,26]. In terms of fresh and dry yield, the current work demonstrated that lower-yielding genotypes (e.g., Mizuna and Pak Choi) present higher increments of yield increase from the first to the second true-leaf stage compared to the faster-growing and higher-yielding genotypes (e.g., Komatsuna and Mibuna). Delaying harvest of these slower-growing genotypes until the appearance of the second true leaf may allow for significant return in yield (e.g., 80.1% in Mizuna) but this practice presently reduced crop turn-over by 41.7% owing to the increase in crop cycle from 7 to 12 days. Therefore, the decision to harvest at S2 instead of S1 must be analyzed in terms of yield for each genotype, with harvesting at S2 seeming economically sound for low-yielding genotypes.

### 4.2. Mineral Composition

Minerals are essential components of the human diet for preventing nutritional disorders and facilitating metabolic and homeostatic regulation through their multiple functionalities [36]. Fresh fruits and vegetables contribute approximately 35, 24, 11 and 7% of the human dietary intake in macrominerals K, Mg, P, and Ca, respectively [37]. With respect to the mineral composition of microgreens, the present study is largely in agreement with previous ones that defined K, Ca, P and Mg as the most abundant macrominerals and Mn, Fe and Zn as the most abundant microminerals [2,7,12]. Moreover, several researchers have posited that certain macro- and microminerals (Mg, Ca, Mn, Fe, Zn, Mo and Se) may be found in higher concentrations in microgreens compared to the full developmental stage of the same genotypes [4,21,24]. An additional advantage claimed for microgreens is their higher mineral bioavailability owing to the suppression of phytate concentration during germination [25,38]. However, the capacity for uptake and accumulation of minerals varies widely with genotype, as presently demonstrated for genotypes of the *Brassicaceae* and previously for other genotypes [15,16]. Although nutrient supplementation and growth conditions may modulate the mineral content of microgreens Renna et al. [17], the current study highlighted genotype selection as a key factor for designing microgreens of particular mineral profiles; e.g., Pak Choi and Komatsuna yielded microgreens high in P and K, Mibuna yielded Ca-rich and Se-rich microgreens and Mizuna Mo-rich microgreens. From a nutritive aspect, it is also noteworthy that the Na/K ratio, high levels of which are implicated in cardiovascular stress [39], varies widely with microgreens genotypes as it was presently found three-fold higher in Mizuna and Pak Choi compared to Komatsuna and Mibuna. 

The current study moreover highlighted that developmental changes in the mineral profiles of microgreens differ according to mineral element and genotype. Calcium concentration consistently increased in all species, which might relate to the increasing demands for mechanical support in the developing microgreens. However, P and Na concentrations and that of most microminerals (Mo, Se and Zn) was nonresponsive to the growth stage. Macro-minerals K and Mg varied between growth stages according to genotype, with a prevailing trend for decrease in K and increase in Mg concentration from S1 to S2. Analogous findings were reported by Waterland et al. [24], who found higher mineral content on a dry weight basis in kale microgreens than in adult kale leaves. They found higher K concentration at the cotyledonary stage than the two-leaf stage but no change in K, P, Mg and Ca and most microminerals between the two-leaf and baby-leaf (four-leaf) stages. However, on a fresh weight basis, they found maximum mineral content at the baby-leaf (four to six leaves) stage compared to both microgreens and adult leaves. 

Pinto et al. [21], on the other hand, reported mineral concentrations on a fresh weight basis and found lettuce microgreens lower in N, P, K and Ca but higher in Fe, Mn, Zn, Mo and Se than their adult counterparts. Waterland et al. [24] highlighted that discrepancies in mineral concentrations may derive from variation in water content between genotypes and developmental stages, reporting decrease in water content up to 15% between microgreens and adult leaves. Although lower water content (higher dry matter) was presently also observed between stages S1 and S2, the nominal difference was limited (0.5%) and does not justify the variation observed mainly in macrominerals. Limited variation in water as opposed to mineral content was also reported for lettuce microgreens and adult leaves by Pinto et al. [21] who reported higher content of most minerals (Ca, Mg, Fe, Mn, Zn, Se and Mo) in microgreens than mature leaves. In the context of the present study, it may be inferred that mineral content on a dry weight basis presents wider differences between genotypes than between growth stages. Variation between growth stages is negligible for most microminerals whereas for Ca, K, Mg, Mn and Fe it depends on genotype and cannot be attributed mainly to increasing dry matter content. The present findings enrich the rather limited reference base for microgreens mineral content.

### 4.3. Antioxidant Capacity

The antioxidant radical-quenching activity may vary significantly between leafy genotypes, especially between red and green ones, with the antioxidant capacity of the former being generally higher [40], hence their inclusion in the human diet is expected to counter the impact of oxidative stress. The antioxidant value of colored microgreens has been posited by previous workers [41], and is further corroborated by the present study where the purple microgreens of Komatsuna and Pak Choi demonstrated higher in vitro antioxidant capacity than the green Mizuna and Mibuna. Moreover, the antioxidant value of microgreens seems more variable by genotype than growth stage. Although the antioxidant capacity of certain species (e.g., Komatsuna and Mizuna) may be higher at the first-leaf stage, this phenomenon cannot be generalized since in other genotypes (e.g., Pak Choi and Mibuna) differences in antioxidant capacity from the first to the second-leaf stage tend to be nonsignificant.

Lutein and β-carotene are the key hydrophobic carotenoid molecules found in microgreens that have been shown to exercise lipophilic antioxidant capacity owing to the light-absorbing and ROS-quenching properties of their conjugated double bond-rich polyene chains [42]. Lutein has thus been administered as a nutritional supplement for ophthalmic protection against short wavelength radiation and light-induced macular damage [43]. Biological activity is also demonstrated by β-carotene that constitutes a precursor of vitamin A, which is essential for immune and ophthalmic functions [42]. Both carotenoid molecules appear more variable across genotypes than between the first and second-true leaf stages of ontogeny, with β-carotene having showed no response to the growth stage whereas lutein concentration demonstrated a genotype-specific increase at the appearance of the second leaf. It is therefore questionable whether delaying harvest to this stage would significantly benefit the bioactive value of microgreens derived from carotenoids. A predominantly genotype-specific variability across growth stages also seems to be the prevailing pattern for the concentration of chlorophyll in microgreens. Similar findings highlighting the significant cultivar-growth stage interaction for chlorophyll and carotenoid pigments were previously demonstrated for red and green butterhead lettuce cultivars harvested at the microgreens and mature growth stages [22]. Contrarily, Klopsch et al. [23] and Heinze et al. [44] reported a significant increase of chlorophylls and carotenoids from the cotyledonary microgreens to mature-leaf stage. Genotype-growth stage interaction aside, it may be inferred that the brief interval in ontogeny from S1 to S2 may confound variation in these pigments that constitute central components of the developing photosynthetic apparatus biosynthesized according to the growing light-harvesting requirements [45].

Overall, the ascorbate concentration (73.8–119.3 mg kg^−1^ fw) obtained in the four brassicaceous genotypes of the present study is in the low end of the range reported in the available literature on microgreens [25,46,47]. Although higher ascorbate content is generally expected in microgreens than sprouts, owing to microgreens’ longer photosynthetic activity that provides hexose products necessary for ascorbate biosynthesis [48], this difference is not always pronounced at close stages of ontogeny. Developmental variation in microgreens ascorbic acid concentration exhibits a strongly genotype-dependent effect with nonsignificant change in some genotypes (e.g., Mibuna) as opposed to others. Moreover, variation along developmental stages is not uniform across genotypes, with increase from S1 to S2 being the common response in most genotypes but decrease also being observed in others (e.g., Pak Choi). The current observation of cultivar-growth stage interaction for ascorbate concentration in brassicaceous microgreens corroborates the findings of Di Bella et al. [6] in their comparative study of sprouts, microgreens and baby leaves of two *Brassica oleracea* L. cultivars and those of El Nakhel et al. [22] on butterhead lettuce microgreens vs. mature leaves.

### 4.4. Volatile Organic Compounds (VOCs)

The brassicaceous microgreens VOCs profile is dominated by isothiocyanate compounds that constitute catabolic products of glucosinolate metabolism. In the present study, this is suggested by the balanced patterns in the relative abundance of key isothiocyanates observed in different genotypes and growth stages. For instance, 3-butenyl isothiocyanate and allyl isothiocyanate varied inversely between genotypes, the former being found in higher relative abundance at the first-leaf stage and the latter at the second-leaf stage. Changes in the profile and concentration of glucosinolates and their catabolic products have been demonstrated in different *Brassicaceae* genotypes and stages of ontogeny, with both categories generally found in higher concentrations at the microgreens stage compared to the mature-leaf stage [44]. Furthermore, the glucosinolates and their breakdown products have been shown to differ in relative abundance and complexity with progressive ontogeny of brassicaceous genotypes, with more abundant and diverse products formed in Pak Choi microgreens than kale microgreens [23]. The relative abundance of the major VOCs presently identified varied between genotypes more extensively than between growth stages. Analogous variation in the total isothiocyanates and their bioaccessible fraction was demonstrated for different brassicaceous microgreens by de la Fuente et al. [25]. Moreover, response to the growth stage was not uniform across genotypes. It is worth noting however that the dominant volatile aromatic component in the microgreens volatile profile (3-butenyl isothiocyanate) had higher relative abundance at S1 than S2, whereas the reverse trend was dominant for the rest of the identified VOCs. This suggests that 3-butenyl isothiocyanate fueled the production of glucosinolate catabolites with an enhanced presence at S2 and corroborates previous studies reporting higher content of glucosinolate catabolites at earlier stages of ontogeny [23].

Another noteworthy change observed at the second-leaf stage is that the cyclic monoterpene limonene, which imparts a characteristic citrus-like aroma, increased in all genotypes during transition from S1 to S2. Similar increase at S2 was also observed for the aldehydes nonanal, which imparts a sweet floral (orange-flower-like) scent, and for trans-2-hexenal, which imparts a fresh leafy green scent (The Good Scents Company, 2021). Therefore, the development of more distinctive aroma profiles during transition from S1 to S2 in brassicaceous microgreens may be inferred. However, these changes cannot be termed universal since the volatile profile of certain genotypes seems less prone to developmental change, with Mizuna microgreens as a current example.

### 4.5. Anthocyanins

Microgreens are a specialty crop that has become a popular gastronomic ingredient owing, among others, to their vivid colors [1]. Anthocyanins are responsible for the purple, blue and red pigmentation of microgreens. However, their role in plants extends to biological functions such as ROS scavenging, protection from radiation and other stress conditions as well as to defense against pathogens [49,50]. The bioactive value of anthocyanins for human health has also been posited, hence the consumption of colored greens and fruits is encouraged [51]. The anthocyanin concentrations in the green genotypes Mibuna and Mizuna presently studied were shown not to change in response to the growth stage. However, developmental changes in the red genotypes were inconsistent as anthocyanin concentration increased in Komatsuna during transition to S2 as opposed to Pak Choi where maximal concentrations were observed at S1. Moreover, only certain anthocyanin molecules demonstrated significant positive correlation with the hydrophilic antioxidant capacity of microgreens extracts, whereas other molecules and the total anthocyanins concentration did not (data not shown). This is not surprising as the in vitro antioxidant capacity of naturally occurring anthocyanins in the juice or aqueous extracts of pigmented fruits, vegetables and flowers is not consistent across genotypes; moreover, although it has been documented in vitro for several genotypes, analogous in vivo activity has not been widely demonstrated with respect to human consumption [52]. It is therefore apparent that apart from the clear divide in the relative abundance of anthocyanins between green- and purple-colored microgreens, variation with developmental stage is largely genotype-specific and the choice of harvest stage based on visual quality must be defined on a genotype-by-genotype case.

### 4.6. Polyphenols

Flavonol glycosides, hydroxycinnamic acids and their derivatives were the dominant classes of phenols present in the four brassicaceous microgreens examined. The current findings are largely consistent with previous studies on brassicaceous microgreens that identified glycosides of quercetin and kaempferol as the main flavonoid glycosides present [23], and the derivatives of caffeic and ferulic acids among the main hydroxycinnamic acids found in conjugation with sugars or other hydroxycinnamic acid moieties [53,54,55]. Despite broad qualitative similarities in their phenolic composition, quantitative differences between genotypes were wide, confirming analogous findings on other brassicaceous microgreens [25]. It is further apparent that the natural pigmentation of microgreens is not a sound indication of their phenolic content. Although anthocyanins comprise a class of high-molecular-weight glycosylated polyphenols, their abundance in pigmented microgreens does not constitute a criterion for their content in total polyphenols, as presently demonstrated by the higher phenolic content of the green microgreens Mibuna and Mizuna compared to the purple microgreens Komatsuna and Pak Choi. The current study further highlighted that genotype is potentially a far more significant source of variation in phenolic content than growth stage, especially in the narrow window of ontogeny presently examined. 

The phenolic composition at the cotyledonary (rootless sprouts), microgreen (first true leaf) and baby-leaf (third true leaf) stages of three Brassica genotypes was examined by Di Bella et al. [6] who identified a general tendency for declining phenolic content, with the content being more often higher in sprouts vs. microgreens than in microgreens vs. baby leaves. However, similarly to the current findings, this trend was underlined by genotype × growth stage interaction rendering the effect of ontogeny at the early post-germination stages largely genotype-dependent. Analogous genotype × growth stage interaction was reported for different amaranth genotypes harvested at the sprout and microgreen stages [47]. It is known that the germination process drives major events in the biosynthesis and metabolism of polyphenols through the phenylpropanoid pathway that may generate differences between successive stages of ontogeny [56]. The absence of significant growth stage effect observed on several of the 20 phenolic components presently identified is consistent with the findings of Di Bella et al. [6] that post-germination differences in phenolic composition between first-leaf and third-leaf microgreens (or baby leaves) tend to be minimal although both stages are characterized by higher phenolic content than the mature leaves of the corresponding genotypes [22,23].

## 5. Conclusions

The ontogenetic stages for harvesting microgreens range from the cotyledonary stage to the emergence of the second true leaf. In the current study, the mineral content of brassicaceous microgreens presented wider differences between genotypes than growth stages S1 and S2. Variation in most microminerals with growth stage was negligible whereas for Ca, K, Mg, Mn and Fe it was genotype-dependent. Antioxidant capacity was in certain genotypes higher at S1 but growth stage differences were nonsignificant in other genotypes. Key carotenoids lutein and β-carotene varied widely by genotype whereas β-carotene showed no response to the growth stage and lutein a genotype-dependent increase at S2. It is therefore questionable whether delaying harvest to S2 would significantly benefit the bioactive value of microgreens derived from carotenoids. 

Ascorbate concentration also exhibited genotype-dependent variation with growth stage, with nonsignificant change found in certain genotypes (e.g., Mibuna). The volatile components of brassicaceous microgreens comprised mostly isothiocyanate catabolites of glucosinolate metabolism, the relative abundance of which varied mostly by genotype, with certain genotypes (e.g., Mizuna) seeming less prone to developmental changes. The current study further highlighted that against the narrow window of ontogeny examined, genotype is the principal source of variation for microgreens’ phenolic content. The absence of significant growth stage effect on many of the phenolic components identified is consistent with previous findings that post-germination differences in phenolic composition between S1 microgreens and baby leaves are minimal. Finally, lower-yielding genotypes register higher relative increase in fresh yield between S1 and S2, compared to faster-growing and higher-yielding genotypes. Although the optimal harvest stage must be determined for each genotype considering yield increase against crop turnover reduction, harvesting at S2 seems advisable for the lower-yielding genotypes. 

## Figures and Tables

**Table 1:** Yield and colorimetric attributes of four *Brassica rapa* L. microgreens genotypes harvested at the first (S1) or second true-leaf stage (S2). All data are expressed as mean ± standard error, *n* = 3.

Source of Variance	Yield	Dry Weight	Dry Matter	Chroma
	(kg FW m^−2^)	(g m^−2^)	(%)	
Genotype (M)				
Komatsuna	2.65 ± 0.14 ^a^	157 ± 14 ^a^	5.89 ± 0.21 ^b^	4.61 ± 0.33 ^c^
Mibuna	2.63 ± 0.16 ^a^	165 ± 12 ^a^	6.27 ± 0.09 ^a^	23.58 ± 0.94 ^b^
Mizuna	2.19 ± 0.28 ^c^	120 ± 20 ^c^	5.35 ± 0.24 ^c^	33.29 ± 1.05 ^a^
Pak Choi	2.45 ± 0.16 ^b^	136 ± 11 ^b^	5.54 ± 0.12 ^c^	4.26 ± 0.79 ^c^
*p*-value	<0.001	<0.001	<0.001	<0.001
Growth stage (S)				
S1	2.07 ± 0.10 ^b^	113 ± 7.5 ^b^	5.43 ± 0.15 ^b^	14.74 ± 3.61
S2	2.89 ± 0.03 ^a^	176 ± 4.6 ^a^	6.09 ± 0.09 ^a^	18.13 ± 3.92
*p*-value	<0.001	<0.001	<0.001	0.051
M × S				
Komatsuna × S1	2.34 ± 0.05 ^b^	127 ± 4.5 ^ef^	5.42 ± 0.08 ^cd^	3.96 ± 0.04 ^f^
Komatsuna × S2	2.95 ± 0.09 ^a^	188 ± 7.1 ^ab^	6.35 ± 0.06 ^a^	5.26 ± 0.33 ^e^
Mibuna × S1	2.27 ± 0.08 ^b^	140 ± 4.5 ^de^	6.17 ± 0.03 ^ab^	21.52 ± 0.16 ^d^
Mibuna × S2	2.99 ± 0.03 ^a^	190 ± 6.3 ^a^	6.37 ± 0.17 ^a^	25.65 ± 0.34 ^c^
Mizuna × S1	1.56 ± 0.08 ^c^	75.2 ± 4.6 ^g^	4.82 ± 0.06 ^e^	30.95 ± 0.15 ^b^
Mizuna × S2	2.81 ± 0.06 ^a^	165 ± 6.1 ^bc^	5.88 ± 0.10 ^b^	35.64 ± 0.17 ^a^
Pak Choi × S1	2.10 ± 0.04 ^b^	111 ± 3.0 ^f^	5.31 ± 0.11 ^d^	2.54 ± 0.19 ^g^
Pak Choi × S2	2.80 ± 0.03 ^a^	161 ± 1.4 ^cd^	5.77 ± 0.08 ^bc^	5.98 ± 0.29 ^e^
*p*-value	<0.001	0.005	0.002	<0.001

Different letters within each column indicate significant differences according to a Tukey–Kramer HSD test (*p* = 0.05). Microgreens growth stages were compared according to Student’s *t*-test.

**Table 2:** Mineral content of four *Brassica rapa* L. microgreens genotypes harvested at the first (S1) or second true-leaf stage (S2). All data are expressed as mean ± standard error, *n* = 3. All data are expressed as mean ± standard error, *n* = 3.

Source of Variance	P	K	Ca	Mg	Na	Fe	Mn	Mo	Se	Zn
	(mg g^−1^ DW)	(mg g^−1^ DW)	(mg g^−1^ DW)	(mg g^−1^ DW)	(mg g^−1^ DW)	(μg g^−1^ DW)	(μg g^−1^ DW)	(μg g^−1^ DW)	(μg g^−1^ DW)	(μg g^−1^ DW)
Genotype (M)										
Komatsuna	10.30 ± 0.2 ^a^	16.83 ± 0.6 ^a^	10.28 ± 0.4 ^c^	4.71 ± 0.1 ^ab^	2.75 ± 0.1 ^b^	134 ± 5.2 ^b^	189 ± 19.6 ^b^	8.05 ± 0.3 ^b^	2.32 ± 0.1 ^ab^	102 ± 1.8 ^bc^
Mibuna	7.64 ± 0.2 ^b^	11.92 ± 0.4 ^b^	12.38 ± 0.4 ^a^	4.52 ± 0.2 ^b^	2.04 ± 0.1 ^c^	138 ± 6.7 ^b^	233 ± 29.3 ^a^	10.33 ± 0.4 ^a^	2.73 ± 0.1 ^a^	108 ± 3.0 ^b^
Mizuna	7.16 ± 0.2 ^b^	12.80 ± 0.4 ^b^	11.79 ± 0.2 ^ab^	4.35 ± 0.1 ^b^	3.22 ± 0.2 ^a^	115 ± 2.5 ^c^	194 ± 23.6 ^b^	11.72 ± 0.8 ^a^	2.53 ± 0.1 ^ab^	96 ± 5.1 ^c^
Pak Choi	10.58 ± 0.2 ^a^	17.08 ± 0.4 ^a^	11.28 ± 0.6 ^b^	5.05 ± 0.1 ^a^	2.78 ± 0.1 ^b^	180 ± 6.3 ^a^	210 ± 28.1 ^ab^	10.19 ± 0.6 ^a^	2.02 ± 0.3 ^b^	141 ± 2.9 ^a^
*p*-value	<0.001	<0.001	<0.001	<0.001	<0.001	<0.001	<0.001	<0.001	0.014	<0.001
Growth stage (S)										
S1	9.02 ± 0.4	15.23 ± 0.8	10.70 ± 0.3 ^b^	4.43 ± 0.1 ^b^	2.89 ± 0.2	142 ± 9.3	152 ± 4.0 ^b^	10.70 ± 0.6	2.54 ± 0.1	109 ± 6.7
S2	8.82 ± 0.5	14.09 ± 0.7	12.17 ± 0.3 ^a^	4.88 ± 0.1 ^a^	2.51 ± 0.1	142 ± 6.5	261 ± 9.6 ^a^	9.44 ± 0.4	2.26 ± 0.1	115 ± 4.5
*p*-value	0.338	0.057	0.002	0.002	0.051	0.920	<0.001	0.054	0.168	0.120
M × S										
Komatsuna × S1	10.51 ± 0.1	18.19 ± 0.1 ^a^	9.72 ± 0.4 ^d^	4.64 ± 0.1 ^bcd^	2.98 ± 0.0	143 ± 6.1 ^cd^	146 ± 5.9	8.16 ± 0.3	2.28 ± 0.1 ^ab^	100 ± 1.1 ^bc^
Komatsuna × S2	10.08 ± 0.3	15.48 ± 0.2 ^bc^	10.83 ± 0.5 ^cd^	4.78 ± 0.1 ^abc^	2.53 ± 0.1	125 ± 4.9 ^de^	232 ± 3.2	7.93 ± 0.6	2.36 ± 0.1 ^a^	103 ± 3.4 ^b^
Mibuna × S1	7.58 ± 0.3	12.08 ± 0.4 ^d^	11.58 ± 0.1 ^bc^	4.05 ± 0.1 ^d^	2.11 ± 0.1	123 ± 1.3 ^de^	172 ± 6.3	10.29 ± 0.7	2.86 ± 0.1 ^a^	106 ± 5.9 ^b^
Mibuna × S2	7.70 ± 0.2	11.75 ± 0.7 ^d^	13.19 ± 0.3 ^a^	4.98 ± 0.1 ^ab^	1.97 ± 0.1	153 ± 0.8 ^bc^	295 ± 21.6	10.38 ± 0.5	2.61 ± 0.1 ^a^	110 ± 2.6 ^b^
Mizuna × S1	7.53 ± 0.1	13.59 ± 0.4 ^cd^	11.56 ± 0.3 ^bc^	4.24 ± 0.1 ^cd^	3.55 ± 0.2	111 ± 1.5 ^e^	143 ± 4.1	13.15 ± 0.9	2.47 ± 0.1 ^a^	85 ± 3.4 ^c^
Mizuna × S2	6.79 ± 0.1	12.00 ± 0.4 ^d^	12.03 ± 0.2 ^abc^	4.47 ± 0.2 ^bcd^	2.89 ± 0.2	120 ± 2.8 ^de^	245 ± 13.9	10.29 ± 0.4	2.58 ± 0.1 ^a^	106 ± 2.3 ^b^
Pak Choi × S1	10.45 ± 0.2	17.05 ± 0.7 ^ab^	9.93 ± 0.2 ^d^	4.80 ± 0.2 ^abc^	2.91 ± 0.2	190 ± 8.4 ^a^	149 ± 3.0	11.22 ± 0.5	2.57 ± 0.4 ^a^	143 ± 4.1 ^a^
Pak Choi × S2	10.72 ± 0.2	17.11 ± 0.6 ^ab^	12.64 ± 0.2 ^ab^	5.30 ± 0.1 ^a^	2.65 ± 0.1	171 ± 6.1 ^ab^	272 ± 12.6	9.16 ± 0.5	1.47 ± 0.2 ^b^	139 ± 4.7 ^a^
*p*-value	0.125	0.008	0.015	0.040	0.200	<0.001	0.128	0.970	0.023	0.012

Different letters within each column indicate significant differences according to a Tukey–Kramer HSD test (*p* = 0.05). Microgreens growth stages were compared according to Student’s *t*-test.

**Table 3:** Hydrophillic Antioxidant Capacity (HAC), ascorbic acid, total chlorophyll, lutein and β-carotene contents of four *Brassica rapa* L. microgreens harvested at the first (S1) or second true-leaf stage (S2). All data are expressed as mean ± standard error, *n* = 3.

Source of Variance	HAC	Ascorbic Acid	Total Chlorophyll	Lutein	β-Carotene
	(mmol Ascorbate eq. kg^−1^ DW)	(mg kg^−1^ FW)	(μg kg^−1^ FW)	(mg kg^−1^ DW)	(mg kg^−1^ DW)
Genotype (M)					
Komatsuna	26.07 ± 0.8 ^a^	106.2 ± 5.3 ^b^	953 ± 22.3 ^a^	195.8 ± 3.5 ^b^	461.0 ± 9.3 ^b^
Mibuna	23.41 ± 0.2 ^b^	104.0 ± 1.7 ^b^	567 ± 43.8 ^b^	251.1 ± 19.6 ^a^	502.7 ± 15.0 ^a^
Mizuna	21.07 ± 1.3 ^c^	119.3 ± 4.8 ^a^	551 ± 22.7 ^b^	124.6 ± 6.7 ^c^	308.9 ± 10.3 ^c^
Pak Choi	26.41 ± 0.4 ^a^	73.8 ± 10.6 ^c^	960 ± 19.3 ^a^	203.1 ± 8.3 ^b^	339.5 ± 9.7 ^c^
*p*-value	<0.001	<0.001	<0.001	<0.001	<0.001
Growth stage (S)					
S1	25.07 ± 0.6	102.7 ± 1.9	799 ± 51.8	177.9 ± 10.8	388.8 ± 25.6
S2	23.40 ± 1.0	98.9 ± 9.2	717 ± 70.4	209.3 ± 18.0	417.2 ± 24.8
*p*-value	0.070	0.662	0.052	0.065	0.055
M × S					
Komatsuna ×S1	27.45 ± 1.0 ^a^	96.8 ± 1.4 ^c^	956 ± 28.0 ^a^	192.4 ± 6.2 ^b^	461.4 ± 19.7
Komatsuna × S2	24.69 ± 0.4 ^bc^	115.6 ± 7.2 ^ab^	949 ± 41.0 ^a^	199.1 ± 3.3 ^b^	460.5 ± 6.8
Mibuna × S1	23.21 ± 0.4 ^c^	107.6 ± 1.0 ^bc^	665 ± 2.4 ^b^	211.8 ± 5.4 ^b^	478.5 ± 17.0
Mibuna × S2	23.61 ± 0.2 ^c^	100.3 ± 0.6 ^bc^	470 ± 7.7 ^d^	290.3 ± 18.6 ^a^	526.9 ± 15.9
Mizuna × S1	23.74 ± 1.0 ^c^	109.3 ± 1.8 ^bc^	598 ± 7.7 ^bc^	120.9 ± 11.5 ^c^	293.9 ± 17.1
Mizuna × S2	18.39 ± 0.9 ^d^	129.3 ± 3.3 ^a^	503 ± 15.9 ^cd^	128.2 ± 9.0 ^c^	323.8 ± 4.2
Pak Choi × S1	25.89 ± 0.4 ^ab^	97.1 ± 2.7 ^c^	974 ± 36.6 ^a^	186.6 ± 4.7 ^b^	321.4 ± 8.1
Pak Choi × S2	26.93 ± 0.5 ^a^	50.6 ± 3.4 ^d^	946 ± 18.1 ^a^	219.5 ± 7.0 ^b^	357.5 ± 9.0
*p*-value	<0.001	<0.001	0.009	0.007	0.269

Different letters within each column indicate significant differences according to a Tukey–Kramer HSD test (*p* = 0.05).

**Table 4:** Volatile Organic Compounds (VOCs) content of four *Brassica rapa* L. microgreens harvested at the first (S1) or second true-leaf stage (S2). All data are expressed as mean ± standard error, *n* = 3. All data are expressed as mean ± standard error, *n* = 3.

Source of Variance	Trans-2-Hexenal	3-Butenyl Isothiocyanate	Limonene	Allyl Isothiocyanate	Nonanal	Phenethyl Isothiocyanate
	Relative Percentage Content (%)					
Genotype (M)						
Komatsuna	1.26 ± 0.37 ^b^	78.17 ± 2.02 ^b^	5.03 ± 0.66 ^a^	10.83 ± 1.13 ^b^	1.48 ± 0.17 ^b^	0.89 ± 0.19 ^bc^
Mibuna	0.11 ± 0.05 ^c^	88.45 ± 1.68 ^a^	2.18 ± 0.97 ^bc^	5.16 ± 0.44 ^d^	1.84 ± 0.56 ^b^	0.38 ± 0.13 ^c^
Mizuna	0.53 ± 0.19 ^c^	68.22 ± 0.60 ^c^	0.70 ± 0.30 ^c^	26.18 ± 0.42 ^a^	1.12 ± 0.23 ^b^	1.64 ± 0.25 ^a^
Pak Choi	2.81 ± 1.12 ^a^	76.38 ± 3.54 ^b^	2.69 ± 0.75 ^b^	7.29 ± 0.69 ^c^	3.36 ± 0.77 ^a^	1.42 ± 0.31 ^ab^
*p*-value	<0.001	<0.001	<0.001	<0.001	<0.001	<0.001
Growth stage (S)						
S1	0.25 ± 0.06 ^b^	81.84 ± 2.51 ^a^	1.45 ± 0.50 ^b^	11.49 ± 2.54	1.10 ± 0.14 ^b^	1.31 ± 0.25
S2	2.11 ± 0.59 ^a^	73.77 ± 2.15 ^b^	3.85 ± 0.62 ^a^	13.24 ± 2.49	2.80 ± 0.45 ^a^	0.85 ± 0.14
*p*-value	0.005	0.023	0.006	0.626	0.002	0.123
M × S						
Komatsuna × S1	0.46 ± 0.08 ^c^	82.63 ± 0.12 ^b^	4.05 ± 0.76 ^a^	8.54 ± 0.61 ^c^	1.25 ± 0.19 ^c^	0.65 ± 0.12 ^b^
Komatsuna × S2	2.06 ± 0.15 ^b^	73.71 ± 0.67 ^c^	6.01 ± 0.81 ^a^	13.11 ± 0.86 ^b^	1.72 ± 0.24 ^bc^	1.13 ± 0.33 ^ab^
Mibuna × S1	0.01 ± 0.01 ^c^	91.77 ± 0.83 ^a^	0.19 ± 0.02 ^b^	5.39 ± 0.41 ^cd^	0.76 ± 0.15 ^c^	0.57 ± 0.22 ^b^
Mibuna × S2	0.21 ± 0.05 ^c^	85.13 ± 1.57 ^b^	4.17 ± 0.86 ^a^	4.94 ± 0.87 ^d^	2.92 ± 0.59 ^b^	0.18 ± 0.04 ^b^
Mizuna × S1	0.12 ± 0.03 ^c^	68.78 ± 0.59 ^d^	0.44 ± 0.33 ^b^	25.93 ± 0.23 ^a^	0.73 ± 0.16 ^c^	2.13 ± 0.26 ^a^
Mizuna × S2	0.94 ± 0.08 ^c^	67.65 ± 1.06 ^d^	0.96 ± 0.53 ^b^	26.44 ± 0.87 ^a^	1.51 ± 0.30 ^bc^	1.16 ± 0.08 ^ab^
Pak Choi × S1	0.39 ± 0.05 ^c^	84.17 ± 0.69 ^b^	1.11 ± 0.22 ^b^	6.08 ± 0.14 ^cd^	1.66 ± 0.24 ^bc^	1.91 ± 0.48 ^a^
Pak Choi × S2	5.23 ± 0.60 ^a^	68.58 ± 1.16 ^d^	4.27 ± 0.49 ^a^	8.49 ± 0.96 ^c^	5.06 ± 0.16 ^a^	0.93 ± 0.15 ^ab^
*p*-value	<0.001	<0.001	0.048	0.006	<0.001	0.015

Different letters within each column indicate significant differences according to a Tukey–Kramer HSD test (*p* = 0.05). Microgreens growth stages were compared according to Student’s *t*-test.

**Table 5:** Anthocyanins content of four *Brassica rapa* L. microgreens harvested at the first (S1) or second true-leaf stage (S2). All data are expressed as mean ± standard error, *n* = 3. All data are expressed as mean ± standard error, *n* = 3.

Source of Variance	Cyanidin-3-(caffeoyl)(p-coumaroyl)dihexoside-5-hexoside	Cyanidin-3-(p-coumaroyl)(sinapoyl)dihexoside-5-hexoside	Cyanidin-3-(feruloyl)(sinapoyl)dihexoside-5-hexoside	∑ Anthocyanins
	(μg g^−1^ DW)	(μg g^−1^ DW)	(μg g^−1^ DW)	(μg g^−1^ DW)
Genotype (M)				
Komatsuna	0.43 ± 0.028 ^b^	1.98 ± 0.277 ^b^	8.00 ± 1.22 ^b^	10.41 ± 1.456 ^b^
Mibuna	0.05 ± 0.003 ^c^	0.35 ± 0.024 ^c^	1.96 ± 0.07 ^c^	2.36 ± 0.056 ^c^
Mizuna	0.09 ± 0.008 ^c^	0.07 ± 0.019 ^c^	0.76 ± 0.10 ^c^	0.93 ± 0.116 ^c^
Pak Choi	0.56 ± 0.033 ^a^	4.86 ± 0.382 ^a^	12.79 ± 1.40 ^a^	18.21 ± 1.150 ^a^
*p*-value	<0.001	<0.001	<0.001	<0.001
Growth stage (S)				
S1	0.28 ± 0.065	1.47 ± 0.478	5.84 ± 1.79	7.59 ± 2.315
S2	0.28 ± 0.070	2.17 ± 0.677	5.92 ± 1.36	8.37 ± 2.031
*p*-value	0.947	0.606	0.951	0.538
M × S				
Komatsuna × S1	0.49 ± 0.004 ^b^	1.39 ± 0.137 ^d^	5.30 ± 0.08 ^c^	7.17 ± 0.152 ^c^
Komatsuna × S2	0.37 ± 0.012 ^c^	2.58 ± 0.074 ^c^	10.70 ± 0.31 ^b^	13.66 ± 0.241 ^b^
Mibuna × S1	0.04 ± 0.002 ^d^	0.39 ± 0.027 ^e^	1.90 ± 0.02 ^d^	2.33 ± 0.006 ^d^
Mibuna × S2	0.05 ± 0.001 ^d^	0.31 ± 0.014 ^e^	2.02 ± 0.13 ^d^	2.38 ± 0.122 ^d^
Mizuna × S1	0.09 ± 0.007 ^d^	0.03 ± 0.001 ^e^	0.55 ± 0.06 ^d^	0.67 ± 0.064 ^d^
Mizuna × S2	0.09 ± 0.016 ^d^	0.11 ± 0.004 ^e^	0.97 ± 0.03 ^d^	1.18 ± 0.014 ^d^
Pak Choi × S1	0.50 ± 0.026 ^b^	4.06 ± 0.208 ^b^	15.61 ± 0.75 ^a^	20.17 ± 0.956 ^a^
Pak Choi × S2	0.62 ± 0.035 ^a^	5.66 ± 0.207 ^a^	9.97 ± 1.13 ^b^	16.26 ± 1.365 ^b^
*p*-value	<0.001	<0.001	<0.001	<0.001

Different letters within each column indicate significant differences according to a Tukey–Kramer HSD test (*p* = 0.05).

**Table 6:** Phenolic composition of four *Brassica rapa* L. microgreens harvested at the first (S1) or second true-leaf stage (S2). All data are expressed as mean ± standard error, *n* = 3.

Source of Variance	Quercetin-3-*O*-sophoroside-7-*O*-hexoside	Caffeic Acid	Kaempferol-3-*O*-sophoroside-7-*O*-hexoside	Quercetin-3-*O*-sophoroside	Caffeic Acid Hexoside Isomers	Kaempferol-3-*O*-(caffeoyl)-sophoroside-7-O-hexoside	Quercetin-3-*O*-(feruloyl)-sophoroside-7-O-hexoside	Kaempferol-3-*O*-(coumaroyl) -sophoroside-7-*O*-hexoside	Kaempferol-3-*O*-(synapoyl)-sophoroside-7-*O*-hexoside	Kaempferol-3-*O*-(feruloyl)-sophoroside-7-*O*-hexoside	Coumaroyl Quinic Acid Isomer 1	Naringin	Feruloyl Quinic Acid Isomer	Apigenin-7-*O*-rutinoside	Ferulic acid	Quercetin-3-*O*-glucuronide	Luteolin-3-*O*-rutinoside	Feruloyl glycoside	KAEMPFEROL-3-*O*-rutinoside	Apigenin-7-rhamnoside-4-rutinoside	∑ Phenolic Acids
	(μg g^−1^ DW)	(μg g^−1^ DW)	(μg g^−1^ DW)	(μg g^−1^ DW)	(μg g^−1^ DW)	(μg g^−1^ DW)	(μg g^−1^ DW)	(μg g^−1^ DW)	(μg g^−1^ DW)	(μg g^−1^ DW)	(μg g^−1^ DW)	(μg g^−1^ DW)	(μg g^−1^ DW)	(μg g^−1^ DW)	(μg g^−1^ DW)	(μg g^−1^ DW)	(μg g^−1^ DW)	(μg g^−1^ DW)	(μg g^−1^ DW)	(μg g^−1^ DW)	(μg g^−1^ DW)
Genotype (M)																					
Komatsuna	18.8 ± 2.2 ^a^	2.9 ± 0.5 ^ab^	5.3 ± 0.8 ^c^	5.4 ± 0.2 ^c^	39.0 ± 2.7 ^c^	15.7 ± 0.9 ^c^	28.1 ± 0.5 ^c^	5.1 ± 1.6 ^c^	98.8 ± 11.2 ^b^	17.0 ± 2.0 ^c^	15.2 ± 6.7 ^a^	1.3 ± 0.0 ^c^	7.0 ± 1.1 ^a^	2.4 ± 0.3 ^b^	20.5 ± 3.1 ^d^	252.0 ± 20 ^d^	1.6 ± 0.2 ^c^	79.7 ± 5.2 ^c^	2.1 ± 0.5 ^b^	11.9 ± 3.7 ^b^	630 ± 30 ^d^
Mibuna	15.9 ± 1.3 ^ab^	4.1 ± 0.6 ^ab^	23.1 ± 4.0 ^b^	20.1 ± 0.8 ^a^	74.6 ± 6.8 ^b^	116.7 ± 12.9 ^b^	212.9 ± 9.2 ^a^	18.5 ± 0.8 ^b^	378.2 ± 27.4 ^a^	96.0 ± 8.8 ^b^	1.7 ± 0.7 ^b^	1.5 ± 0.1 ^c^	5.3 ± 0.3 ^b^	5.0 ± 1.2 ^a^	40.0 ± 2.5 ^c^	319.7 ± 13 ^c^	1.3 ± 0.1 ^c^	113.6 ± 4.8 ^b^	2.6 ± 0.5 ^b^	11.6 ± 2.9 ^b^	1462 ± 45 ^b^
Mizuna	10.5 ± 0.4 ^c^	4.4 ± 0.7 ^a^	30.9 ± 2.5 ^a^	8.8 ± 1.0 ^b^	121.9 ± 5.6 ^a^	189.6 ± 10.8 ^a^	155.9 ± 14.7 ^b^	28.3 ± 1.9 ^a^	412.1 ± 27.8 ^a^	167.9 ± 13.5 ^a^	3.4 ± 1.4 ^b^	3.0 ± 0.1 ^a^	3.1 ± 0.2 ^c^	4.5 ± 0.2 ^a^	62.1 ± 4.9 ^a^	484.3 ± 21 ^b^	12.1 ± 1.5 ^a^	196.9 ± 20.5 ^a^	8.6 ± 2.2 ^a^	25.0 ± 3.5 ^a^	1933 ± 61 ^a^
Pak Choi	12.8 ± 1.6 ^bc^	2.6 ± 0.6 ^b^	5 ± 0.4 ^c^	5.8 ± 0.3 ^c^	38.9 ± 2.0 ^c^	14.6 ± 0.6 ^c^	28.3 ± 3.1 ^c^	1.9 ± 0.1 ^d^	81.4 ± 18.8 ^b^	11.3 ± 2.0 ^c^	14.7 ± 6.2 ^a^	2.6 ± 0.2 ^b^	4.9 ± 1.0 ^b^	5.8 ± 0.2 ^a^	48.6 ± 5.0 ^b^	674.8 ± 30 ^a^	2.3 ± 0.2 ^b^	82.8 ± 5.0 ^c^	3.8 ± 0.3 ^b^	10.5 ± 3.3 ^b^	1053 ± 33 ^c^
*p*-value	0.000	0.026	0.000	0.000	0.000	0.000	0.000	0.000	0.000	0.000	0.000	0.000	0.000	0.000	0.000	0.000	0.000	0.000	0.000	0.000	0.000
Growth stage (S)																					
S1	14.1 ± 1.7	2.6 ± 0.4 ^b^	12.7 ± 2.8 ^b^	9.6 ± 1.9	64.8 ± 10.3	80 ± 23.0	97.7 ± 21.7	11.2 ± 3.0 ^b^	287.0 ± 50.3 ^a^	61.3 ± 15.6 ^b^	0.5 ± 0.1 ^b^	1.9 ± 0.2 ^b^	4.1 ± 0.5 ^b^	4.7 ± 0.7	34.4 ± 4.2 ^b^	389.4 ± 47 ^b^	3.3 ± 1.0 ^b^	99.6 ± 10.1 ^b^	5.3 ± 1.5	7.4 ± 1.8 ^b^	1191 ± 141 ^b^
S2	14.8 ± 1.1	4.4 ± 0.3 ^a^	19.4 ± 4.3 ^a^	10.5 ± 1.8	72.4 ± 11.0	88.4 ± 22.8	114.9 ± 27.7	15.7 ± 3.5 ^a^	198.3 ± 42.7 ^b^	84.8 ± 23.3 ^a^	17.0 ± 3.8 ^a^	2.3 ± 0.3 ^a^	6.1 ± 0.6 ^a^	4.2 ± 0.4	51.2 ± 5.1 ^a^	475.9 ± 52 ^a^	5.4 ± 1.8 ^a^	136.9 ± 18.8 ^a^	3.3 ± 0.4	22.1 ± 1.9 ^a^	1348 ± 152 ^a^
*p*-value	0.695	<0.001	0.010	0.272	0.147	0.377	0.093	<0.001	<0.001	0.015	<0.001	<0.001	0.018	0.504	<0.001	<0.001	0.024	0.002	0.183	<0.001	<0.001
M × S																					
Komatsuna × S1	22.9 ± 1.8 ^a^	2.4 ± 0.8	3.8 ± 0.6	5.2 ± 0.3 ^c^	42.4 ± 2.7 ^c^	17.1 ± 1.5 ^d^	29.02 ± 0.3 ^d^	1.5 ± 0.3 ^e^	120.8 ± 12.3	12.7 ± 0.9 ^e^	0.7 ± 0.1 ^b^	1.2 ± 0.1 ^c^	5.1 ± 1.6 ^bcd^	1.7 ± 0.3 ^c^	13.5 ± 0.3 ^f^	208.7 ± 7	1.3 ± 0.1 ^d^	68.5 ± 1.8 ^f^	1.5 ± 0.6 ^b^	3.7 ± 0.1	564 ± 7
Komatsuna × S2	14.7 ± 2.2 ^bc^	3.5 ± 0.4	6.9 ± 0.5	5.6 ± 0.2 ^c^	35.6 ± 4.4 ^c^	14.3 ± 0.4 ^d^	27.1 ± 0.6 ^d^	8.6 ± 0.1 ^d^	76.9 ± 1.0	21.2 ± 0.3 ^e^	29.7 ± 3.6 ^a^	1.4 ± 0.1 ^c^	8.8 ± 0.5 ^a^	3.1 ± 0.1 ^bc^	27.5 ± 0.9 ^e^	295.4 ± 6	1.9 ± 0.2 ^cd^	90.8 ± 2.7 ^de^	2.6 ± 0.9 ^b^	20.0 ± 0.3	696 ± 15
Mibuna × S1	13.3 ± 1.2 ^bc^	2.7 ± 0.1	16.4 ± 1.1	20.5 ± 0.6 ^a^	59.8 ± 1.6 ^c^	90.8 ± 7.2 ^c^	200.8 ± 11.7 ^ab^	17.2 ± 0.9 ^c^	431 ± 23.8	78.6 ± 2.5 ^d^	0.1 ± 0.1 ^b^	1.3 ± 0.1 ^c^	5.8 ± 0.2 ^abc^	6.9 ± 1.7 ^a^	34.7 ± 1.2 ^d^	293.3 ± 9	1.1 ± 0.1 ^d^	104.7 ± 4.7 ^cd^	2.6 ± 0.5 ^b^	5.3 ± 1.0	1387 ± 33
Mibuna × S2	18.4 ± 0.7 ^ab^	5.4 ± 0.1	29.7 ± 5.8	19.7 ± 1.6 ^a^	89.4 ± 2.8 ^b^	142.7 ± 10.4 ^b^	225.1 ± 11.6 ^a^	19.9 ± 0.7 ^c^	325.5 ± 20.3	113.5 ± 8.7 ^c^	3.3 ± 0.1 ^b^	1.6 ± 0.1 ^c^	4.8 ± 0.3 ^bcd^	3.0 ± 0.1 ^bc^	45.2 ± 1.3 ^c^	346.1 ± 9	1.5 ± 0.1 ^d^	122.4 ± 3.9 ^c^	2.6 ± 1.0 ^b^	18.0 ± 0.9	1538 ± 59
Mizuna × S1	9.9 ± 0.6 ^c^	3.8 ± 1.4	25.8 ± 2.6	6.7 ± 0.3 ^c^	120.2 ± 11.9 ^a^	197.3 ± 22.4 ^a^	126.8 ± 11.5 ^c^	24.4 ± 0.5 ^b^	472.9 ± 13.0	138.3 ± 5.7 ^b^	0.3 ± 0.1 ^b^	2.8 ± 0.1 ^a^	2.7 ± 0.1 ^cd^	4.2 ± 0.2 ^abc^	51.8 ± 3.3 ^c^	440.7 ± 12	8.8 ± 0.4 ^b^	151.8 ± 4.5 ^b^	13.5 ± 0.6 ^a^	17.5 ± 1.2	1820 ± 70
Mizuna × S2	11.1 ± 0.4 ^c^	5 ± 0.3	35.9 ± 0.2	11 ± 0.1 ^b^	123.6 ± 3.0 ^a^	182 ± 5.0 ^ab^	184.9 ± 10.3 ^b^	32.3 ± 1.2 ^a^	351.3 ± 2.6	197.6 ± 2.2 ^a^	6.4 ± 0.5 ^b^	3.2 ± 0.1 ^a^	3.5 ± 0.3 ^cd^	4.9 ± 0.1 ^ab^	72.4 ± 0.4 ^a^	527.9 ± 11	15.5 ± 0.1 ^a^	242.0 ± 7.3 ^a^	3.6 ± 0.5 ^b^	32.6 ± 1.1	2047 ± 31
Pak Choi × S1	10.4 ± 1.1 ^c^	1.5 ± 0.2	4.7 ± 0.5	6.1 ± 0.3 ^c^	36.6 ± 2.5 ^c^	14.7 ± 0.5 ^d^	34.1 ± 3.3 ^d^	1.7 ± 0.1 ^e^	123.3 ± 1.8	15.6 ± 1.0 ^e^	1.0 ± 0.1 ^b^	2.2 ± 0.1 ^b^	2.7 ± 0.2 ^d^	5.8 ± 0.2 ^ab^	37.5 ± 0.5 ^d^	615.1 ± 25	1.9 ± 0.1 ^cd^	73.3 ± 2.7 ^ef^	3.5 ± 0.5 ^b^	3.1 ± 0.1	995 ± 37
Pak Choi × S2	15.1 ± 2.3 ^bc^	3.7 ± 0.5	5.2 ± 0.7	5.6 ± 0.5 ^c^	41.2 ± 2.9 ^c^	14.6 ± 1.2 ^d^	22.5 ± 2.2 ^d^	2.0 ± 0.1 ^e^	39.5 ± 0.8	6.9 ± 0.7 ^e^	28.4 ± 1.5 ^a^	3.1 ± 0.1 ^a^	7.2 ± 0.1 ^ab^	5.7 ± 0.3 ^ab^	59.7 ± 0.8 ^b^	734.4 ± 15	2.7 ± 0.1 ^c^	92.3 ± 5.1 ^de^	4.2 ± 0.2 ^b^	17.9 ± 0.9	1112 ± 27
*p*-value	<0.001	0.423	0.073	0.005	0.012	0.005	0.002	<0.001	0.059	<0.001	<0.001	0.005	0.002	0.004	0.005	0.155	<0.001	<0.001	<0.001	0.211	0.511

Different letters within each column indicate significant differences according to a Tukey–Kramer HSD test (*p* = 0.05). Microgreens growth stages were compared according to Student’s *t*-test.

## Data Availability

The datasets generated for this study are available on request to the corresponding author.

## Supplementary Materials

The following are available online at https://www.mdpi.com/article/10.3390/foods10051032/s1, Figure S1: Representative HPLC-Diode array chromatogram of carotenoids extracted from Komatsuna microgreens monitored at 450 nm, Figure S2: UHPLC-HRMS chromatogram of anthocyanin ex-tracted from Komatsuna microgreens, Figure S3: UHPLC-HRMS chromatogram of polyphenolic compounds extracted from Komatsuna microgreens, Figure S4: Representative chromatograms of VOCs extracted from Komatsuna and Pak Choi microgreens, Table S1: Linearity, limit of detection (LOD), limit of quantification (LOQ), precision and recovery for the eight authentic standards (n = 5).
