# Supplementary material for: Ontogenetic Variation in the Mineral, Phytochemical and Yield Attributes of Brassicaceous Microgreens
Source: Foods. 2021 May 10;10(5):1032. doi: 10.3390/foods10051032 (PMC8151805; doi:10.3390/foods10051032)
Supplement: Supplementary file 1 [file foods-10-01032-s001.zip › foods-1210490-supplementary.pdf]

Table S1. Linearity, limit of detection (LOD), limit of quantification (LOQ), precision and recovery for the eight authentic standards (n = 5).

Compound	LOQ	LOD	Linearity (r2)	Recovery (%) (n = 3)			Intra-Day Precision (RSD, %; (n = 3)	Inter-Day Precision (RSD, %; (n = 3)
	(ng/g)	(ng/g)		1 mg*kg^{-1}	10 mg*kg^{-1}	50 mg*kg^{-1}	5	6
Cyanidin-glucoside	0.04	0.12	0.998	91.5	92.4	99.3	3	8
Rutin	0.05	0.14	0.976	93.6	94.6	91.4	6	9
Kaempferol-3-O-glucoside	0.04	0.12	0.999	93.4	90.1	89.2	7	10
Caffeic acid	0.05	0.14	0.982	96.6	96.9	98.6	5	5
Ferulic acid	0.03	0.10	0.991	98.5	93.7	92.7	4	7
Apigenin-rutinoside	0.05	0.14	0.993	99.5	98.3	98.5	2	8
Naringin	0.04	0.12	0.999	95.9	95.8	98.4	7	9
Luteolin-3-O-rutinoside	0.03	0.10	0.993	98.3	89.4	93.5	5	7

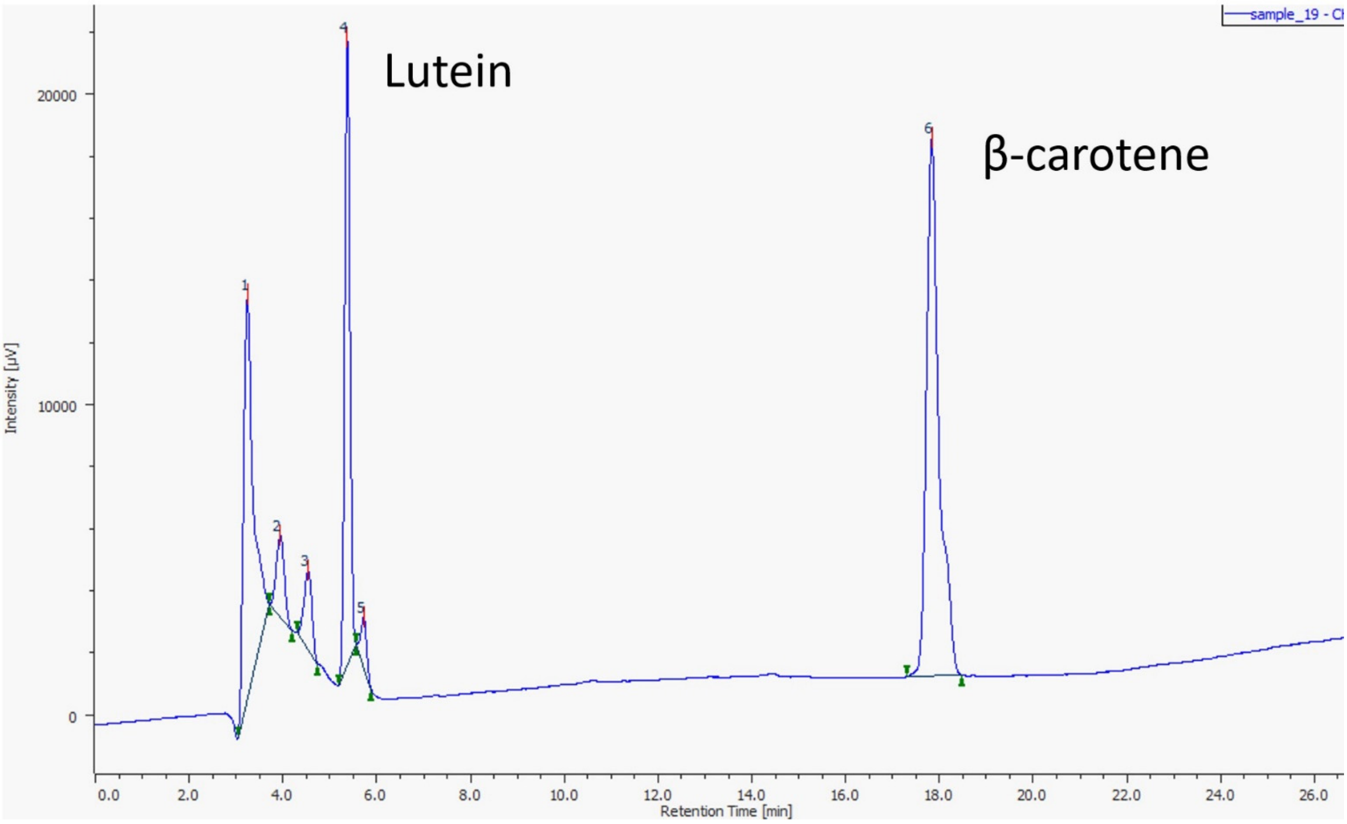

Figure S1. Representative HPLC-Diode array chromatogram of carotenoids extracted from Komatsuna microgreens monitored at 450 nm.

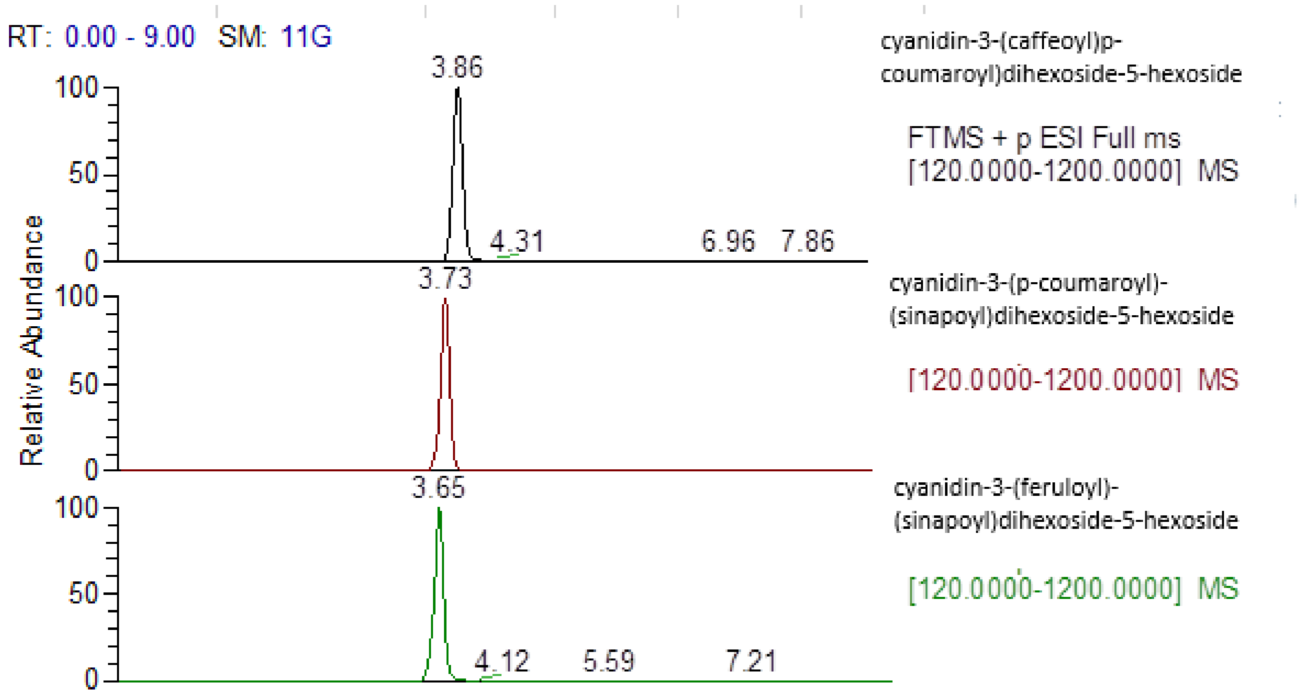

Figure S2. UHPLC-HRMS chromatogram of anthocyanin extracted from Komatsuna microgreens.

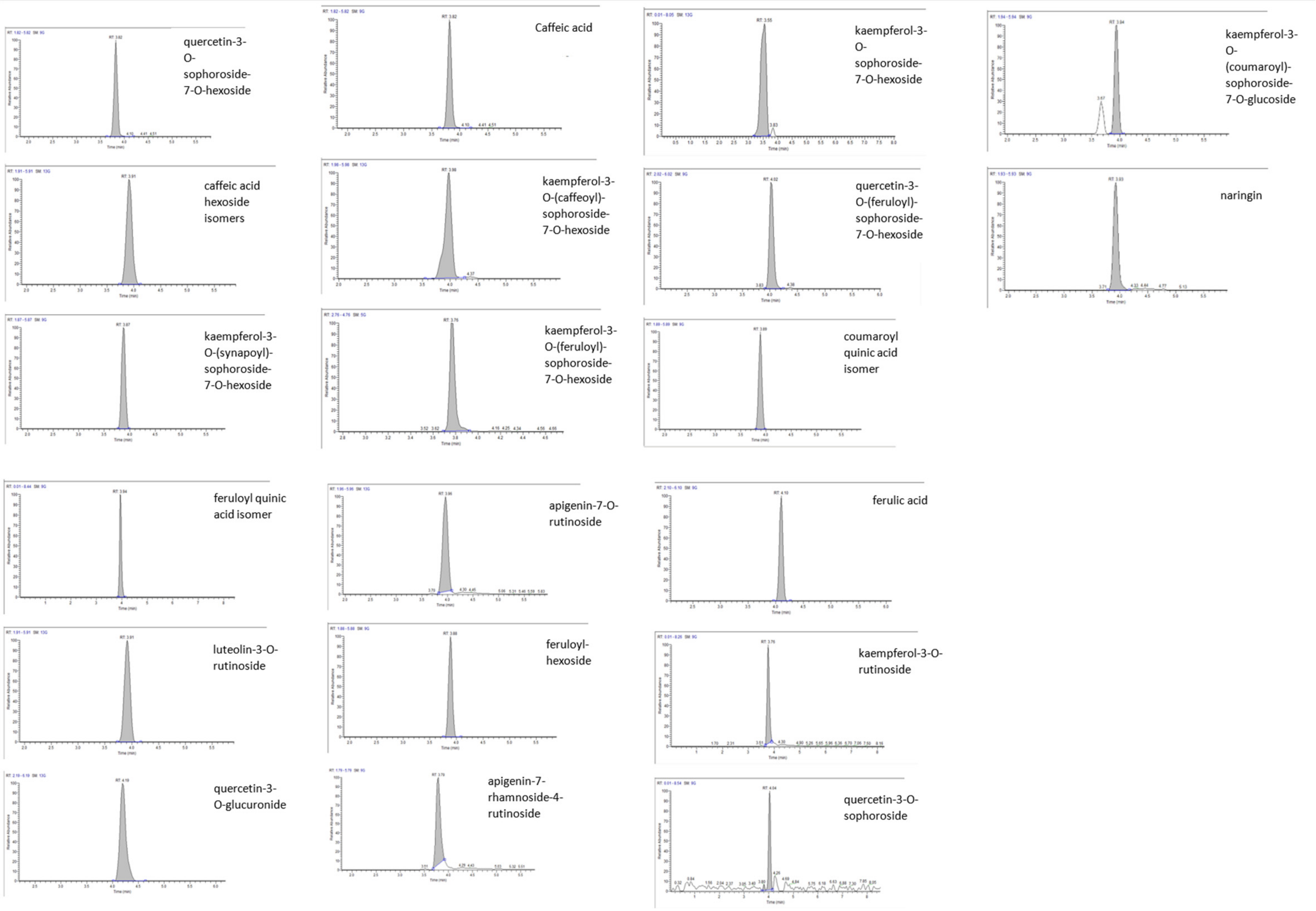

Figure S3. UHPLC-HRMS chromatogram of polyphenolic compounds extracted from Komatsuna microgreens.

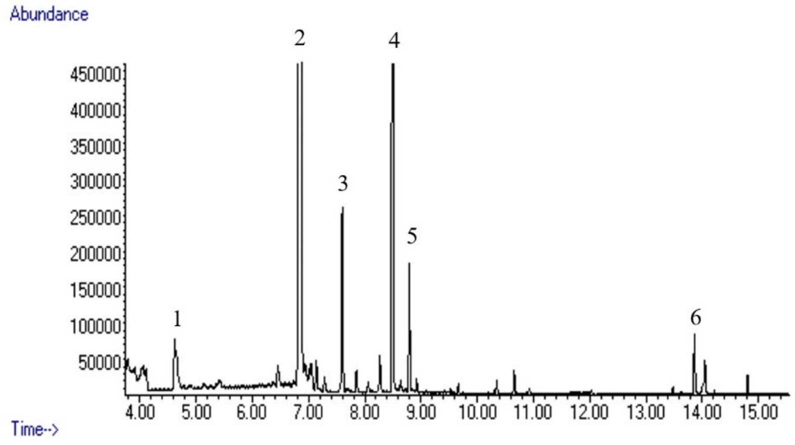

Sample: Komatsuna

Peak	Compound
1	trans-2-Hexenal
2	3-Butenyl isothiocyanate
3	Limonene
4	Allyl Isothiocyanate
5	Nonanal
6	Phenethyl isothiocyanate

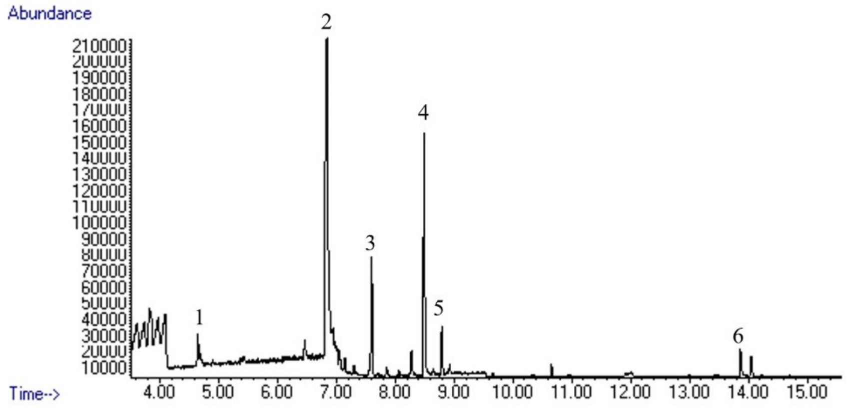

Sample: Pak choi

Peak	Compound
1	trans-2-Hexenal
2	3-Butenyl isothiocyanate
3	Limonene
4	Allyl Isothiocyanate
5	Nonanal
6	Phenethyl isothiocyanate

Figure S4. Representative chromatograms of VOCs extracted from Komatsuna and Pak Choi microgreens.
